# Anomalous Origin of the Right Vertebral Artery from the Right Common Carotid Artery

**DOI:** 10.7759/cureus.3602

**Published:** 2018-11-16

**Authors:** Aamir Ali, Neda I Sedora Roman, Mougnyan Cox, David Kung, Omar Choudhri, Robert W Hurst, Bryan A Pukenas

**Affiliations:** 1 Cardiology, Beth Israel Deaconess Medical Center, Boston, USA; 2 Radiology, Hospital of the University of Pennsylvania, Philadelphia, USA; 3 Neurosurgery, Hospital of the University of Pennsylvania, Philadelphia, USA; 4 Neurosurgery, Hospital of University of the Pennsylvania, Philadelphia, USA

**Keywords:** anomalous origin, right vertebral artery, down syndrome, aberrant right subclavian artery

## Abstract

The anomalous origin of the right vertebral artery (VA) from the right common carotid artery (CCA) is a rare vascular anomaly, which is usually clinically asymptomatic and found incidentally during angiographic examinations. This anomaly is invariably associated with an aberrant right subclavian artery (RSCA). Approximately 31 cases have been reported in the literature. We present a case of a right VA originating from the right CCA in a patient with Down syndrome and discuss the imaging findings, embryological etiology of the anomaly, as well as its implications for endovascular/surgical treatment.

## Introduction

The anomalous origin of the right vertebral artery (VA) from the right common carotid artery (CCA) is a rare vascular variant; approximately 31 cases have been reported in the literature [1-8]. Most of the cases of anomalous origin of VA are incidentally discovered during angiographic or postmortem examinations. Despite being clinically asymptomatic, this anomaly carries diagnostic importance during the preoperative planning of vascular surgery in the neck and for road mapping endovascular approaches to cervical or intracranial pathology [9]. Misadventures in the common carotid artery in these patients carry a risk of ischemic injury to the anterior and posterior circulation since both the internal carotid artery and vertebral artery are supplied by a single vessel. Furthermore, if the VA origin is not identified, patients can be misdiagnosed with VA hypoplasia or aplasia [10]. Studies have shown that if the VA arises from the right CCA, there is also invariably an increased association with an aberrant right subclavian artery (RSCA) [11-12]. Conversely, Tsai et al. showed that among pediatric patients with a known aberrant RSCA, 15.7% had VA anomalies [10]. We present a case of a right VA originating from the right CCA in a patient with Down syndrome and discuss the imaging findings as well as the embryologic etiology of the anomaly.

## Case presentation

**Figure 1:**
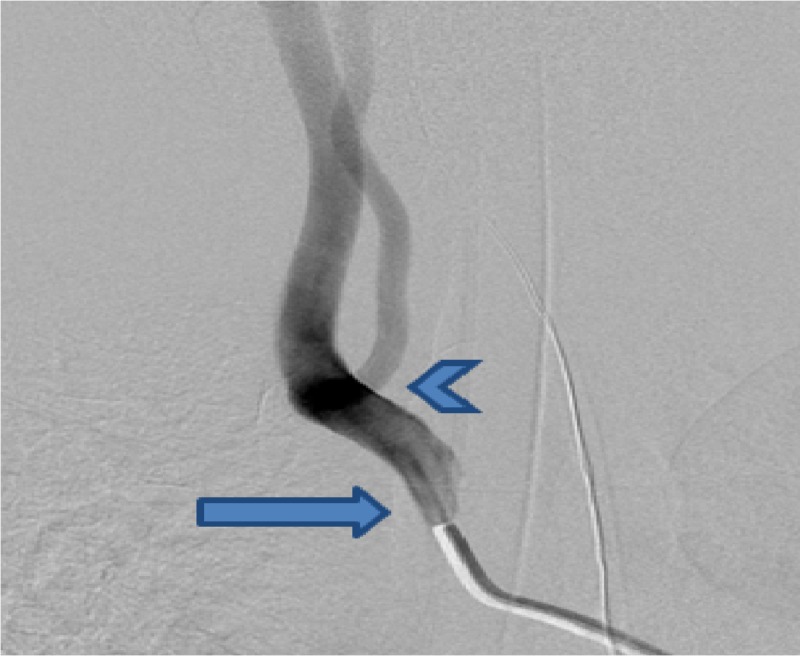
Right common carotid artery injection in the frontal view showing a direct origin of the right vertebral artery (arrowhead) just distal from the origin of the right common carotid artery (arrow)

**Figure 2:**
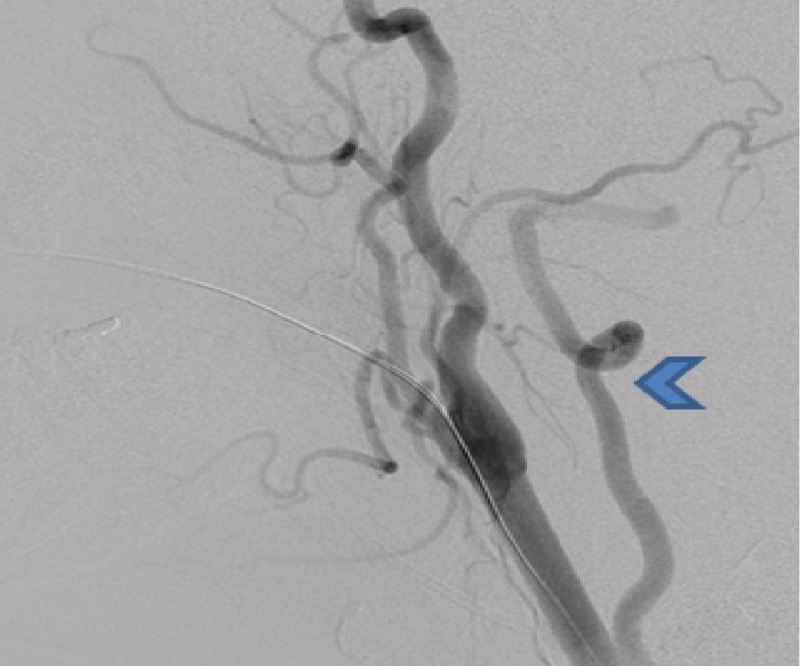
Lateral view of the right common carotid artery injection shows the direct origin of the right vertebral artery (arrowhead) distal from the origin of the right common carotid artery

**Figure 3:**
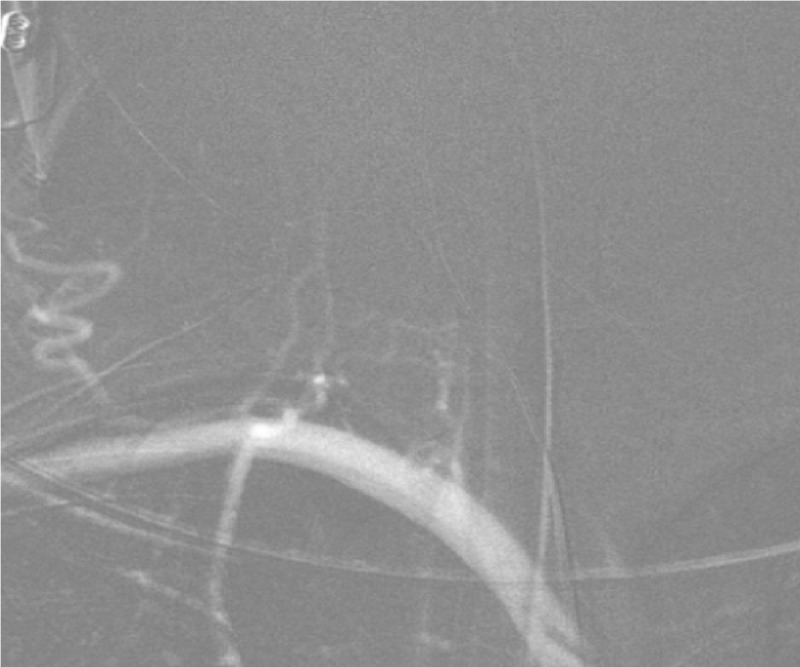
The right subclavian artery roadmap injection in the frontal view shows the absence of a takeoff of the right vertebral artery from the right subclavian artery

A 49-year-old woman with Trisomy 21 had incidental bilateral internal cerebral artery (ICA) aneurysms identified on head computed tomography angiography (CTA) during the workup of new memory loss. Dedicated cerebral angiography was recommended to further characterize the ICA aneurysms. Anteroposterior (AP) and lateral angiographic runs through the right common carotid artery showed a direct takeoff of the right VA just distal to the origin of RCCA (Figures 1-2), with the frontal view of the right subclavian artery roadmap showing an absence of the origin of the right VA from it (Figure 3). Although not documented on a dedicated aortic arch angiogram, this patient also had an aberrant right subclavian artery.

## Discussion

The incidence of anomalous origin of the right VA from the right CCA is 0.18% [13], whereas the incidence of aberrant RSCA is 1.5% [14]. In order to understand the etiology of these anomalies, it is important to briefly review the normal embryology. Embryologically, the right RSCA develops from the right fourth pharyngeal arch artery, right dorsal aorta, and right seventh cervical intersegmental artery. The caudal part of the right dorsal aorta involutes between the origin of the intersegmental artery and the confluence of the left dorsal aorta. Aberrant RSCA arises when the right pharyngeal arch artery and right dorsal aorta disappear cranial to the right seventh intersegmental artery [15]. This results in the formation of an aberrant RSCA from the seventh intersegmental artery and distal aortic root [16]. The aberrant RSCA arises as the last great vessel of the posterior surface of the proximal descending aorta and courses behind the esophagus to the contralateral side to supply the right upper extremity.

The cervical intersegmental arteries contribute to the development of the VA, which normally originates from the subclavian artery. The cervical intersegmental arteries, also known as ventral segments, arise from the dorsal aorta and connect the dorsal aorta and longitudinal anastomoses [15]. These arteries provide blood supply to the cervical nerves and spinal cord during the early embryonic period. The development of the VA starts with the formation of longitudinal anastomoses between the first seven cervical intersegmental arteries at the 10 to 12 mm stage of the embryo. These anastomoses form the distal part of the VA. The seventh cervical intersegmental artery is attached to the subclavian artery and forms the proximal part of the VA. Involution of the first six ventral segments results in the normal origin of the VA from the subclavian artery. Anomalies arise when these ventral segments persist. The failure of involution of the third through fifth cervical intersegmental arteries results in an anomalous origin of the VA from the CCA [17].

Prior studies have demonstrated a higher incidence of vascular anomalies in patients with Down syndrome; in fact, Rathore et al. showed that the incidence of VA anomalies and aberrant RSCA in patients with Down syndrome is 40% and 36%, respectively [4,18- 19]. The case we report demonstrates both an aberrant RSCA and an anomalous origin of the right VA from the right CCA in a patient with Down syndrome.

## Conclusions

Although the anomalous origin of the right vertebral artery from the right common carotid artery is rare, knowledge of its existence is important during preoperative surgical and endovascular planning. Knowledge of the association of these vascular anomalies in patients with Down syndrome is also useful when treating this patient population.
